# An economic evaluation of expanding hookworm control strategies to target the whole community

**DOI:** 10.1186/s13071-015-1187-5

**Published:** 2015-11-05

**Authors:** Hugo C. Turner, James E. Truscott, Alison A. Bettis, Kathryn V. Shuford, Julia C. Dunn, T. Déirdre Hollingsworth, Simon J. Brooker, Roy M. Anderson

**Affiliations:** London Centre for Neglected Tropical Disease Research, London, UK; Department of Infectious Disease Epidemiology, School of Public Health, Faculty of Medicine, St Marys Campus, Imperial College London, Norfolk Place, London, W2 1PG UK; Mathematics Institute, University of Warwick, Coventry, CV4 7AL UK; School of Life Sciences, University of Warwick, Coventry, CV4 7AL UK; Faculty of Infectious and Tropical Diseases, London School of Hygiene and Tropical Medicine, London, UK

**Keywords:** Hookworm, Modelling, Cost-effectiveness analysis, Preventative chemotherapy, Community-wide treatment

## Abstract

**Background:**

The WHO treatment guidelines for the soil-transmitted helminths (STH) focus on targeting children for the control of morbidity induced by heavy infections. However, unlike the other STHs, the majority of hookworm infections are harboured by adults. This untreated burden may have important implications for controlling both hookworm’s morbidity and transmission. This is particularly significant in the context of the increased interest in investigating STH elimination strategies.

**Methods:**

We used a deterministic STH transmission model and parameter estimates derived from field epidemiological studies to evaluate the impact of child-targeted (2–14 year olds) versus community-wide treatment against hookworm in terms of preventing morbidity and the timeframe for breaking transmission. Furthermore, we investigated how mass treatment may influence the long-term programmatic costs of preventive chemotherapy for hookworm.

**Results:**

The model projected that a large proportion of the overall morbidity due to hookworm was unaffected by the current child-targeted strategy. Furthermore, driving worm burdens to levels low enough to potentially break transmission was only possible when using community-wide treatment. Due to these projected reductions in programme duration, it was possible for community-wide treatment to generate cost savings – even if it notably increases the annual distribution costs.

**Conclusions:**

Community-wide treatment is notably more cost-effective for controlling hookworm’s morbidity and transmission than the current child-targeted strategies and could even be cost-saving in many settings in the longer term. These calculations suggest that it is not optimum to treat using the same treatment strategies as other STH. Hookworm morbidity and transmission control require community-wide treatment.

**Electronic supplementary material:**

The online version of this article (doi:10.1186/s13071-015-1187-5) contains supplementary material, which is available to authorized users.

## Background

The most common neglected tropical diseases (NTDs) are the soil-transmitted helminths (STH) which include *Ascaris lumbricoides, Trichuris trichiura* and the hookworms (*Ancylostoma duodenale* and *Necator americanus*). In the 2010 Global Burden of Disease study, the hookworms were estimated to be responsible for over 65 % of the total disability-adjusted life years (DALY) lost due to STH infections [1] and, worldwide, are the second leading cause of anaemia [2].

**Fig. 1:**
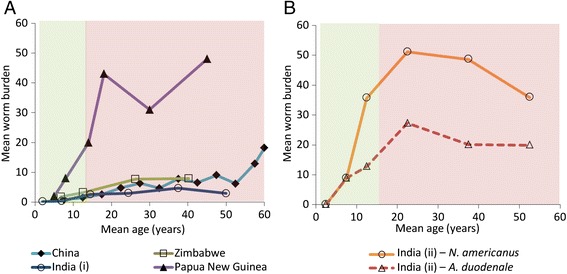
The reported relationships between host age and mean worm burden for hookworm: The data is from the following worm expulsion studies; India (i, Tamil Nadu (Vairavankuppam)) [40], Papua New Guinea [41], Zimbabwe [12], China [42], and India (ii, West Bengal) [43]. Panel **a** illustrates the variation across the different study locations and Panel **b** from the different hookworm species. Children 2–14 (who are targeted for treatment) are shaded in green and adults, (≥15 year olds (who are not targeted for treated) are shaded in red. With the assumed host demography used within the model (from Uganda) [8], the proportion of worms harboured by adults varied between 70 and 85 % for the different profiles

The current WHO guidelines focus on targeting STH treatment to preschool-aged (Pre-SAC: 2–4 year olds) and school-aged children (SAC: 5–14 year olds) with the ultimate goal to “eliminate STH as a public health problem” in children by 2020 (though treatment of women of childbearing age and adults in certain high risk occupations is also recommended where possible) [3, 4]. However, unlike the other STH, the majority of the hookworms are harboured by adults (Fig. 1), as opposed to the children. Consequently, although the current guidelines may be effective in terms of reducing the morbidity of both *Ascaris* and *Trichuris* (where the burden peaks in the targeted children), they may not be as effective against hookworm.

In addition, there has recently been an increasing interest in considering different strategies for breaking the transmission of STH [5–7] since it is widely recognised that in the absence of effective water, sanitation and hygiene (WASH) programmes, childhood treatment alone is unlikely to break transmission. Previous STH modelling studies have illustrated that even in low transmission settings it is not possible to eliminate hookworm with preventative chemotherapy alone when only targeting children [8, 9, 10].

Expanding treatment programmes to include adults through community-wide treatment is under consideration. However, it should be noted that this would be a major shift in STH control policy, and require an increase in resources and international commitment. Consequently, it is important to evaluate how cost-effective expanding control programmes to include adults is, and understand if it could potentially generate long term cost-savings.

The aim of this paper is to investigate the impact, long-term cost and cost-effectiveness of using community-wide treatment for the control of hookworm.

## Method

This analysis was performed with a fully age-structured deterministic model of the dynamics of STH transmission (described in [8–10]), using the parameters pertaining to hookworm (Additional file 1: Table S1). The model can estimate the number of treatment rounds of a given strategy required to theoretically achieve elimination – defined as crossing the breakpoint in transmission where infection levels settle to the equilibrium of extinction [11].

The relative exposure of the different age groups to the infectious reservoir defined in the model was estimated using likelihood methods and the age intensity profile of infection reported by Bradley et al. [12] (Fig. 1). This fit is presented in Additional file 1: Figure S1.

The efficacy of albendazole (400 mg) against hookworm (defined within the model as the proportion of worms expelled per treatment) was assumed to be 94.8 % [13]. In the sensitivity analyses we also considered a much lower efficacy of 64.2 % [14]. Individuals under two years of age were considered ineligible for drug treatment.

### Scenarios and output

The model was used to compare child-targeted annual and biannual treatment (targeting Pre-SAC and SAC, 2–14 year olds [3, 4]) to annual community-wide treatment (targeting ≥2 year olds). Two different transmission settings were explored; intermediate (R_0_ = 2.5), and high (R_0_ = 5) – as measured by the basic reproductive number (R_0_) which defines the average number of female worm offspring generated by a single female worm in her lifespan who survive to reproductive maturity in the absence of density-dependent or mate-availability constraints [11]. We considered a range of different coverage levels of adults (35–75 %).

#### Morbidity

The model was used to estimate the proportion of the population at risk of morbidity. It was assumed morbidity arose in those with a heavy infection, defined by an age-specific worm burden above the standard thresholds [15] (Table 1). These thresholds were based on the observed relationship between infection intensity and anaemia [16]. Though we are using a previously established method [15, 17], it is important to acknowledge that the relationships between the intensity of STH infection and morbidity are complex and not well understood and that this is therefore a standard simplifying assumption [15, 18].

**Table 1:** Age-specific worm burden thresholds for morbidity [15]

Age class (years)	Lower threshold	Higher threshold
0–4	20	80
5–9	30	120
10+	40	160

The lower thresholds are based on empirical observations of worm numbers associated with developmental deficits [15, 17]. The higher thresholds are a more conservative value intended to reflect more serious clinical consequences and to provide a lower bound to the estimate of morbidity [15, 17]

#### Costs

Due to the absence of cost data for community-wide STH treatment [19], a range of relative increases in the economic distribution cost (total per year) when using community-wide compared to child-targeted treatment were assumed (+50, +100, +150, and +200 % i.e. if the full economic cost of a child-targeted programme was US$100,000 per year, this would increase to US$150,000, US$200,000, US$250,00 and US$300,000 per year respectively when using community-wide treatment). Though the relative increase in costs were not based on any primary cost data, this wide range would capture a very broad range of settings/scenarios. Due to the notable variation in reported costs of STH control programmes [19], the cost of annual child-targeted treatment was assumed to be arbitrary – as we are only considering the relative increase in cost/cost-effectiveness of community-wide treatment.

As MDA programmes have substantial economies of scale, assuming constant cost per treatment and varying treatment coverage within a model can be highly misleading [20, 21]; as the fixed costs of MDA are not accounted for, this method can notably underestimate the costs of treating at a lower coverage within a given transmission setting. Due to this, we assumed a range of different relative increases in the total cost per year of the treatment programme when using community-wide treatment. This reflects that the increase in cost will vary across different settings due to a range of programmatic factors i.e. it is possible to have a relatively inexpensive programme with a high coverage and an expensive programme with a low coverage.

It was assumed that after elimination was achieved, the distribution cost stopped immediately. This reflects that although using a community-wide treatment strategy will initially increase the programmatic distribution costs, it may generate cost savings compared to using a child-targeted strategy for the full time horizon depending on the reduction in the required programme duration (due to the capacity to break transmission). It is important to note that the potential programmatic costs are only considered within the assumed time horizon for the simulations (the potential cost savings achieved by elimination will be greater the longer the assumed time horizon, which is assumed to be finite in the simulations, but could, in theory, be infinite).

It is also important to note that the costs were considered from the perspective of the health care service provider (since the costs of accessing the intervention are likely to be negligible).

#### Cost-effectiveness analysis

The model was used to estimate the relative increase in cost-effectiveness of community-wide treatment in terms of preventing years lived with morbidity (Table 1). This was done by comparing the incremental cost (based on assumed percentage increase in cost of community-wide treatment) and incremental benefit of community-wide treatment in terms of preventing years lived with morbidity in both adults and children to that of annual child-targeted treatment, as estimated from the modelling.

#### Discounting

Discounting is the process of adjusting the future values of costs to reflect the fact that society prefers to receive benefits sooner and pay costs later. The discount rate reflects the strength of this time reference. As recommended by the WHO [22], a discount rate of 3 % per year was applied to the costs and effects. The sensitivity of the results to the use of different discount rates was also explored [22].

## Results

### Morbidity control

**Fig. 2:**
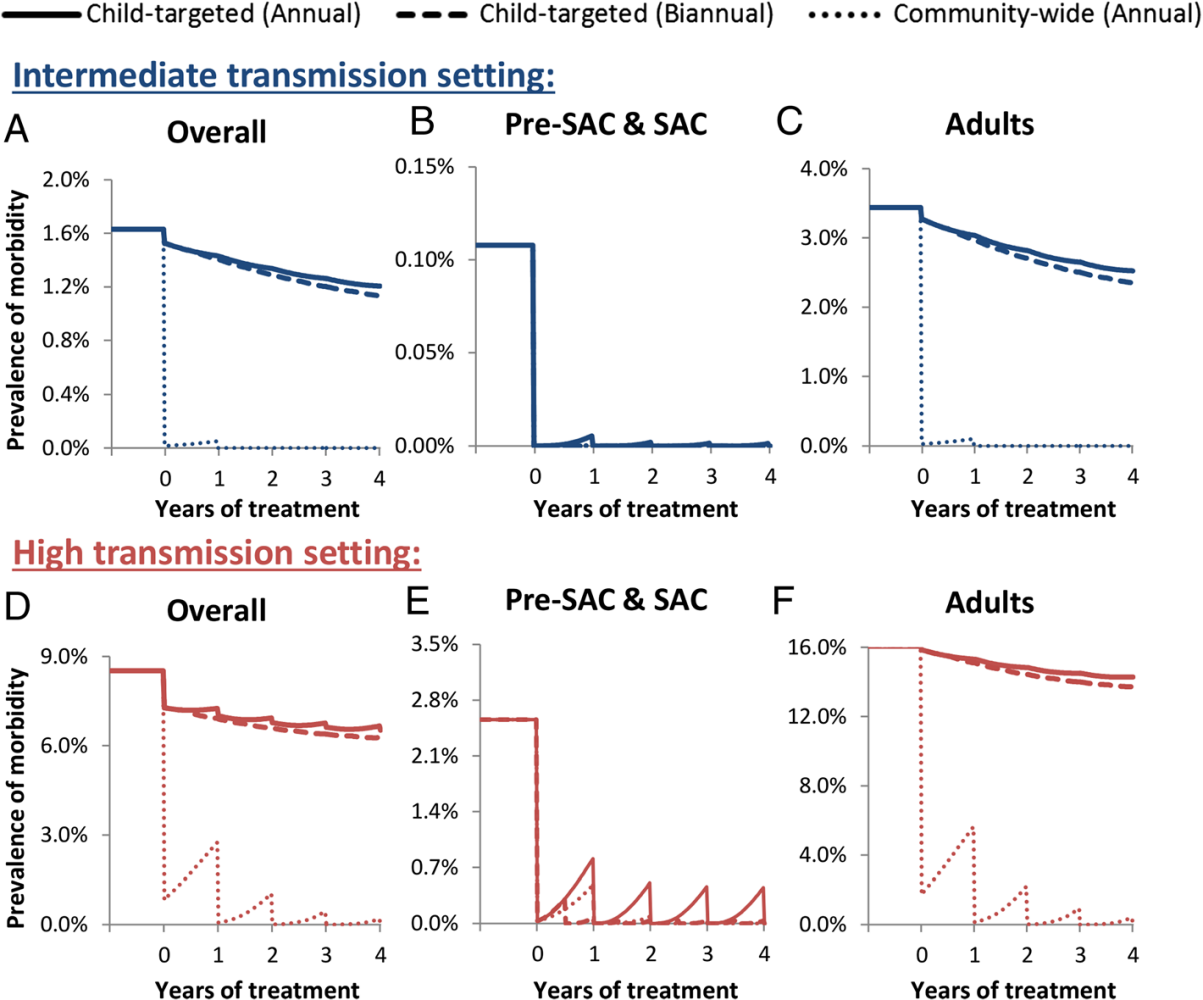
Impact of different treatment strategies on the prevalence of morbidity in (**a**) the overall community, (**b**) Pre-SAC & SAC and (**c**) adults. Two different transmission settings were explored; intermediate (R_0_ = 2.5), and high (R_0_ = 5) – as measured by the basic reproductive number (R_0_) [11]. Panels **a** & **d** illustrate the overall mean number of worms across all ages, panels (**b)** & (**e**), the mean number of worms in children (Pre-SAC and SAC, 2–14 year olds), panels (**c**) & (**f**) the mean number of worms in adults (≥15 year olds). The different styled lines represent different treatment strategies: solid – annual targeted treatment (Pre-SAC and SAC), dashed – biannual targeted treatment (Pre-SAC and SAC), and dotted – annual community-wide treatment (Pre-SAC, SAC and adults). Individuals under two years of age were not eligible for treatment. The results assume 80 % coverage per round of targeted age group(s), and 94.8 % treatment efficacy. The results employ the lower intensity thresholds for morbidity (presented in Table 1). The corresponding results using the higher intensity threshold are presented in Additional file 1: Figure S2

The model projected that the recommended annual child-targeted treatment (2–14 year olds) effectively reduces the prevalence of morbidity in children (Fig. 2) – even in high transmission settings – supporting the WHO guideline suggestions for controlling child morbidity. For adults the worm burden threshold for morbidity is higher than for children (Table 1), and yet the prevalence of morbidity is higher in adults than in children. Consequently, a large proportion of hookworm’s overall disease burden is unaffected by the current treatment strategy – even when achieving a high coverage (Fig. 2). The overall impact of annual child-targeted treatment decreased with the intensity of the transmission setting (Fig. 2).

Increasing the treatment frequency in children (biannual child-targeted treatment) was projected to generate only very small additional reductions in the prevalence of morbidity and still did not affect a large proportion of the overall burden (Fig. 2 and Table 2). In contrast, annual community-wide treatment was projected to be markedly more effective (317–748 % with a 75 % treatment coverage of adults) in reducing the overall disease burden (Table 2) and equivalent to biannual child-targeted treatment in reducing the prevalence of morbidity in children (Fig. 2).

**Table 2:** The number of morbidity case years averted for the three different strategies

Transmission setting	Intensity thresholds for morbidity	Annual child-targeted treatment	Biannual child-targeted treatment (^a^)	Community-wide treatment (^a^)
Intermediate	Lower	370	498 (35 %)	3136 (748 %)
	Higher	2.4	3.2 (33 %)	9.8 (317 %)
High	Lower	1230	1638 (33 %)	6075 (394 %)
	Higher	93	135 (46 %)	583 (527 %)

^a^Percentage gain relative to annual child-targeted treatment. The effect is expressed in terms of morbidity case years prevented i.e. the number of years lived with morbidity averted across the time horizon. For each year the effectiveness was calculated as the difference between the number of cases and the number in absence of treatment. The results assume 75 % coverage per round of targeted age group(s), a total population size of 200,000, a discount rate of 3 %, and 94.8 % treatment efficacy. Analysis was conducted with a two-year implementation period and a 15-year time horizon (i.e. looking at the effect of two years of treatment for 15 years). The intensity thresholds for morbidity are presented in Table 1

The results in Fig. 2 pertain to the lower intensity thresholds for morbidity (Table 1). When using the higher intensity threshold the pre-control prevalence of morbidity was notably lower and was almost negligible in the intermediate transmission setting (Additional file 1: Figure S2). Though the number morbidity case-years averted was sensitive to the assumed intensity thresholds for morbidity, the notable relative impact of community-wide treatment was robust (Table 2). The sensitivity of the impact of community-wide treatment to the level of coverage in adults is shown in Table 3. Even at a low coverage of adults (35 %) community-wide treatment still had a noteworthy impact on morbidity (Table 3) (between 168 and 339 % more than child-targeted treatment).

**Table 3:** The sensitivity to the relative increase in the number of morbidity case years averted by annual community-wide treatment to the level of coverage in adults

		Community-wide treatment		
Transmission setting	Intensity thresholds for morbidity	Adult coverage: 75 %	Adult coverage: 55 %	Adult coverage: 35 %
Intermediate	Lower	749 %	520 %	339 %
	Higher	317 %	258 %	203 %
High	Lower	394 %	275 %	168 %
	Higher	527 %	439 %	327 %

Percentage gain is relative to annual child-targeted treatment with a coverage of 75 %. The effect is expressed in terms of morbidity case years prevented i.e. the number of years lived with morbidity averted across the time horizon. For each year the effectiveness was calculated as the difference between the number of cases and the number in absence of treatment. Assumptions are as in Table 2

#### Cost-effectiveness of morbidity control

The cost-effectiveness of community-wide treatment in terms of preventing morbidity was notably higher than annual child-targeted treatment – even when assuming it increases programme distribution costs (Table 4). When assuming a low coverage of adults, the increases in cost-effectiveness were lower for a given increase in cost (Table 4). However, even when the coverage of adults was as low as 35 %, the model projected an increase in cost-effectiveness (with the exception of when the increase in the cost per year was 200 % (Table 4)).

**Table 4:** The increase in the cost-effectiveness of preventing morbidity when using annual community-wide versus child-targeted treatment

Transmission setting	Intensity thresholds for morbidity	Relative increase in the distribution costs (total per year) of community-wide versus child-targeted treatment			
		+50 %	+100 %	+150 %	+200 %
Coverage of adults: 75 %					
Intermediate	Lower	+1397 %	+649 %	+399 %	+274 %
	Higher	+534 %	+217 %	+111 %	+59 %
High	Lower	+688 %	+294 %	+163 %	+97 %
	Higher	+953 %	+427 %	+251 %	+163 %
Coverage of adults: 35 %					
Intermediate	Lower	+577 %	+239 %	+126 %	+69 %
	Higher	+307 %	+103 %	+36 %	+2 %
High	Lower	+236 %	+68 %	+12 %	**−16 %**
	Higher	+554 %	+227 %	+118 %	+63 %

The percentage increase compares the incremental relative cost and incremental benefit of community-wide treatment to that of annual child-targeted treatment (75 % coverage of children). The benefit is expressed in terms of morbidity case years prevented i.e. the number of years lived with morbidity averted. Assumptions are as in Table 2. As the results only investigate the impact of two treatment rounds they do not account for any potential cost savings resulting from breaking transmission with community-wide treatment. Negative values are shown in bold i.e. community-wide treatment was not more cost-effective

When using the higher intensity threshold for morbidity (which corresponds to >160 worms in adults (Table 1)), the relative increase in cost-effectiveness was lower in intermediate transmission settings than for a lower morbidity threshold because the prevalence of these infections was so low in these settings (Table 2 and Additional file 1: Figure S2). Note that the results in Table 4 only investigate the impact of two treatment rounds, and therefore do not account for any potential cost savings resulting from breaking transmission with community-wide treatment – which would increase cost-effectiveness even further.

Even when assuming that the drug efficacy was lower than previously assumed (64.2 % versus 94.8 %), community-wide treatment was still predicted to generate notable benefits and therefore it appears that the general conclusions are robust (Additional file 1: Figure S3).

### Transmission control

**Fig. 3:**
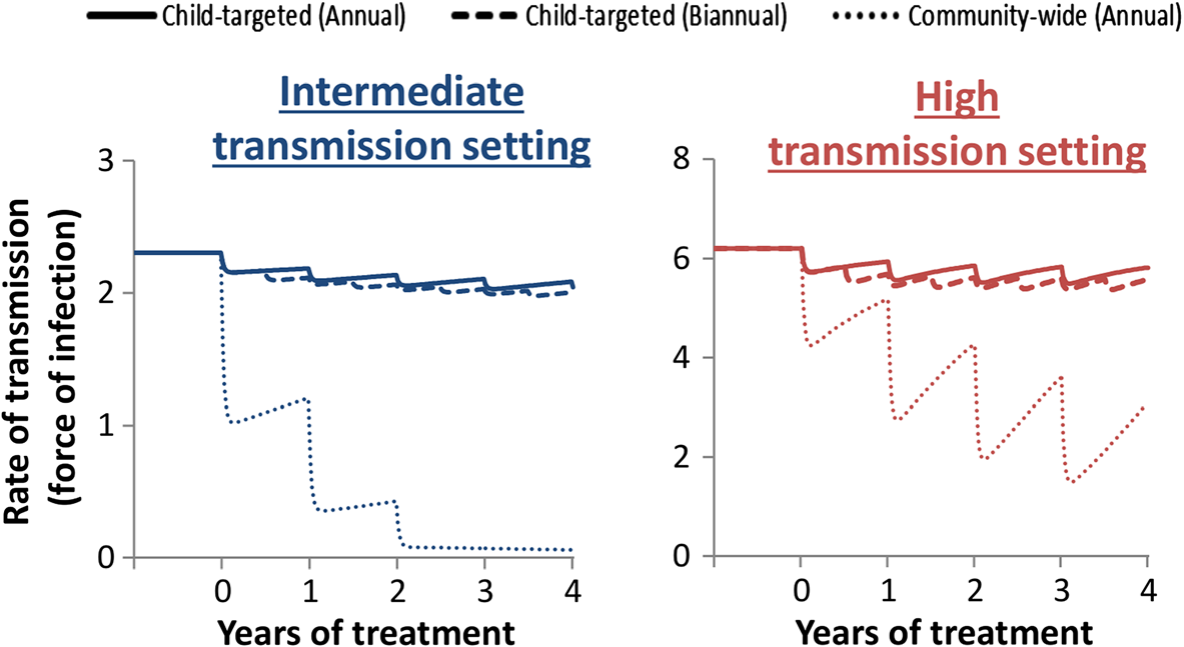
Impact of different treatment strategies on the overall rate of transmission. Force of infection: the mean number of incoming worms per person per year (a metric for the level of on-going transmission within the model). Two different transmission settings were explored; intermediate (R_0_ = 2.5), and high (R_0_ = 5) – as measured by the basic reproductive number (R_0_) [11]. The different styled lines represent different treatment strategies: solid – annual targeted treatment (Pre-SAC and SAC), dashed – biannual targeted treatment (Pre-SAC and SAC), and dotted – annual community-wide treatment (Pre-SAC, SAC and adults). Results assume 80 % coverage per round of target age group(s), and 94.8 % treatment efficacy. Individuals under two years of age were not eligible for treatment

Child-targeted treatment (either annual or biannual) was not found to notably impact the overall level of transmission; children are therefore likely to become reinfected quickly after treatment from the adult reservoirs of infection – even in intermediate transmission settings (Fig. 3). In contrast, community-wide treatment greatly reduces the rate of transmission (because the adults have the majority of the overall worm burden (Fig. 1)). Consequently, treating adults can also benefit the children (Fig. 2) –as it reduces the level of transmission for everyone (Fig. 3). This is the reason that annual community-wide treatment is predicted to have a comparable impact on heavy infections in children as biannual child-targeted treatment (Fig. 2).

#### Feasibility of and timeframe for breaking transmission

**Fig. 4:**
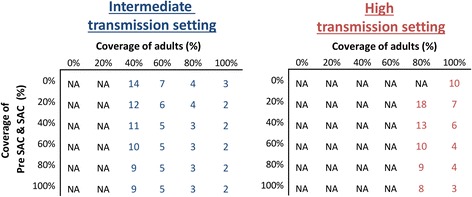
Number of years of annual treatment to achieve elimination of *hookworm* as a function of coverage of children versus adults. Two different transmission settings were explored; intermediate (R_0_ = 2.5), and high (R_0_ = 5)– as measured by the basic reproductive number (R_0_) [11]. Results assume a 94.8 % treatment efficacy. Pre-SAC and SAC, 2–14 year olds; and adults, ≥15 year olds. Individuals under two years of age were not eligible for treatment. NA; Not achievable within 15 years of annual treatment

The level of treatment coverage in adults was the major determinant of the feasibility of eliminating hookworm transmission (Fig. 4). The higher the level of transmission (measured by the R_0_), the harder elimination was projected to be – both in terms of the necessary level of coverage and the required number of annual rounds of treatment to cross the transmission breakpoint [11] (Fig. 4). For example, with an overall coverage of 75 % it was projected to take three rounds to break transmission in the intermediate (R_0_ = 2.5) transmission setting, and nine rounds in the high (R_0_ = 5) (Fig. 4).

#### Total programme costs and cost savings

**Fig. 5:**
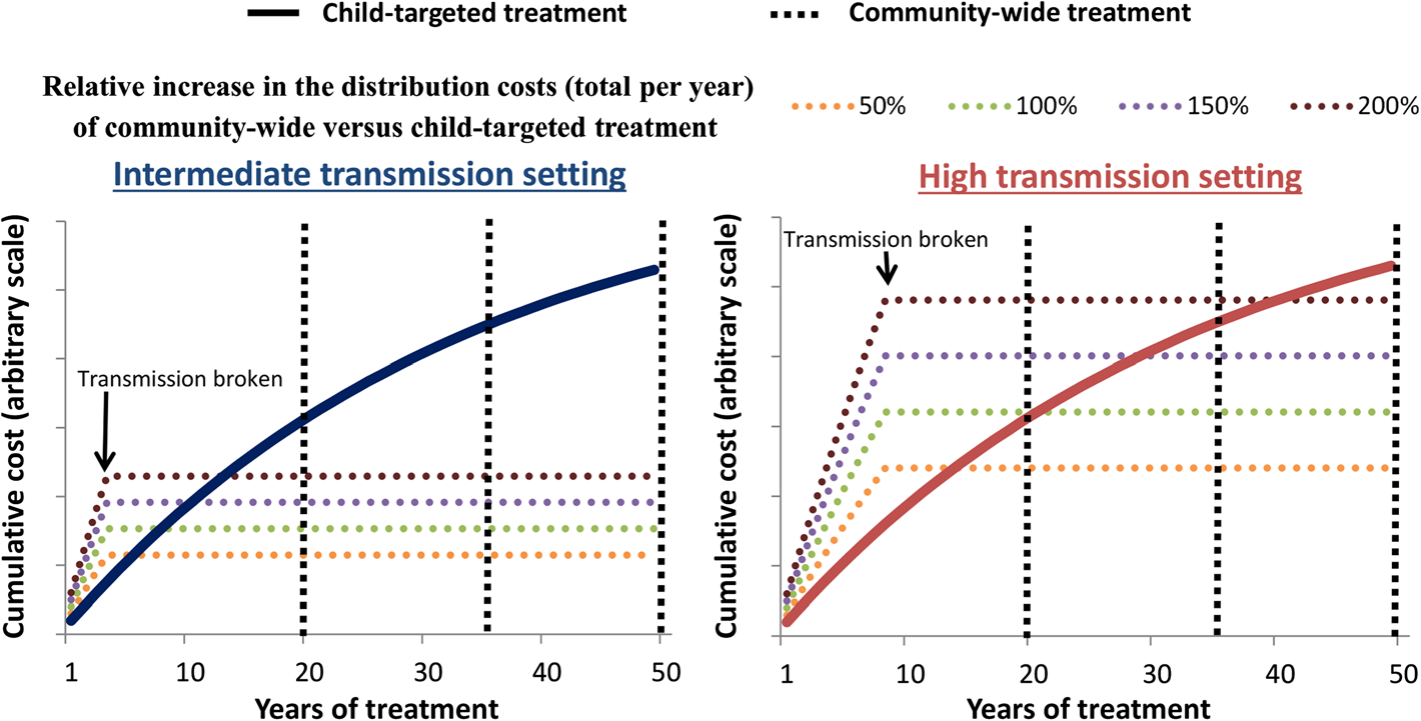
Cumulative total cost of community-wide versus child-targeted treatment. Annual targeted treatment (Pre-SAC and SAC) and annual community-wide treatment are represented by a solid and dashed line respectively. Costs were assumed to cease after elimination is achieved. The coloured dotted lines represent a range of different values regarding the cost per year of distributing community-wide treatment relative to child-targeted treatment. Two different transmission settings were explored; A) intermediate (R_0_ = 2.5), and B) high (R_0_ = 5) – as measured by the basic reproductive number (R_0_) [11]. The results assume 75 % coverage per round of targeted age group(s), and 94.8 % treatment efficacy. Costs were discounted at 3 % per year (resulting in the slope of the curve). The different vertical lines highlight the different employed time horizons. Individuals under two years of age were not eligible for treatment

Community-wide treatment is projected to be able to break transmission, permitting a shorter duration for the treatment programme (Fig. 4). It is therefore possible for it to generate cost savings, compared to the current child-targeted treatment strategy – even when it notably increases the annual distribution costs of the programme (Fig. 5 and Table 5). The overall costs are the annual costs times the number of years of treatment required to cross the transmission breakpoint. The longer the time horizon considered for the analysis, the greater the potential cost savings of community-wide treatment (Fig. 5 and Table 5). The higher the transmission setting, the lower the potential cost savings of community-wide treatment (Fig. 5 and Additional file 1: Table S2). As expected, community-wide treatment was projected to cost more than child-targeted treatment in these settings when using a short time horizon (as this does not account for any longer-term cost saving given elimination of transmission). The point at which community-wide treatment becomes cost-saving was dependent on both the assumed cost of community-wide treatment and the employed time horizon (Fig. 5, Table 5 and Additional file 1: Table S2).

**Table 5:** The relative total cost of annual community-wide versus child-targeted treatment in an intermediate transmission setting

	Relative increase in the distribution costs (total per year) of community-wide versus child-targeted treatment			
	+50 %	+100 %	+150 %	+200 %
Time horizon				
Coverage of adults: 75 %				
20 years	−71 %	−62 %	−52 %	−43 %
35 years	−80 %	−74 %	−67 %	−60 %
50 years	−83 %	−78 %	−72 %	−67 %
Coverage of adults: 55 %				
20 years	−45 %	−27 %	−6 %	**+9 %**
35 years	−62 %	−50 %	−35 %	−24 %
50 years	−68 %	−58 %	−45 %	−37 %
Coverage of adults: 35 %				
20 years	**+1 %**	**+4 %**	**+68 %**	**+101 %**
35 years	−30 %	−7 %	**+16 %**	**+39 %**
50 years	−41 %	−23 %	−3 %	**+16 %**

The results assume an annual treatment strategy, 75 % treatment coverage in children, and 94.8 % treatment efficacy. Costs were discounted at 3 % per year. Results in bold are where the total costs of community-wide treatment are higher than child-targeted treatment (i.e. it was not cost saving and has a higher relative cost). Results for the higher transmission setting are shown in Additional file 1: Table S2

For a given incease in cost, the chance of cost-savings from comminity-wide treatment were smaller the lower the level of coverage achivied in adults (Table 5) – though were notable even if the coverage was relatively low (55 %) (Table 4). When assuming the coverage of adults was 35 %, cost savings were projected to be unlikely without long time horizons for the modelled setting (Table 4) [12].

When assuming a lower drug efficacy of 64.2 %, the number of rounds required to break transmission was higher (Additional file 1: Figure S4) and therefore cost-savings was lower/less likely. With this pessimistic assumption on efficacy, it was not possible to eliminate in high transmission settings with MDA alone (Additional file 1: Figure S4) and therefore no cost savings were projected in this scenario.

The projected timeframes for elimination and cost savings were consistent when using a different parameter for the strength of the density dependence in egg production by female worms (Additional file 1: Table S3) and were even more pronounced when using an alternative dataset for the baseline age profile of infection intensity (particularly for lower coverages) (Fig. 1 and Additional file 1: Table S4).

#### Discounting

The employed discount rate had a major impact on the potential cost savings generated by reaching elimination through community-wide treatment (Additional file 1: Table S5). The higher the discount rate, the lower the potential cost savings (as these savings occur in the future – illustrated by the gradient of the solid curve in Fig. 5). However, even with a high discount rate of 6 %, community-wide treatment could still generate notable cost savings (depending on the time horizon and transmission settings).

## Discussion

### Morbidity control

Our projections indicate that the current STH control strategy, which focuses on targeting children, is ineffective in reducing the overall burden of disease induced by hookworm infection – though it is effective in the context of controlling morbidity in children (Fig. 2). This occurs because the burden of infection lies largely in adults due to the rising burden of infection with age (Fig. 1) [24]. Consequently, the disease burden is projected to be higher in adults compared to children (Fig. 2). This is important because hookworm is estimated to be responsible for over 65 % of the DALYs lost due to STH infections [1], and worldwide it is the second leading cause of anaemia [2]. Our analyses show that in order to control the overall disease burden of hookworm with preventive chemotherapy, programmes must include adults through community-wide treatment (Fig. 2), which is projected to be notably more cost-effective in terms of preventing morbidity (Table 4).

In areas with a STH prevalence greater than 50 %, WHO guidelines recommend that treatment frequency in children is increased to at least twice a year [3]. Though this would have a notable benefit for *Ascaris* and *Trichuris* [8, 23], our calculations indicate that this strategy was projected to generate only very small additional reductions in both morbidity (Fig. 2), and the level of overall transmission (Fig. 3) for hookworm [Turner et al., ‘Analysis of the population-level impact of co-administering ivermectin with albendazole or mebendazole for the control and elimination of Trichuris trichiura’, submitted].

It is important to note that the motivation for focusing on treating children for STH is in part due to the potential developmental consequences of infection in children (though this is an area that needs more investigation [25]) and in part due to the ease with which SAC can be accessed through the school. Though the evidence that intestinal worms directly interfere with productivity and wage earning capacity in adults (reviewed in [26]) is circumstantial, many of the health consequences of hookworm (especially anaemia [27, 28]) have been proven to affect productivity in working teenagers and adults [29]. Consequently, treating adults for hookworm could generate economic gains and accelerate economic development in endemic countries.

Over the past decade, many areas may have received community-wide treatment which impacts STH through lymphatic filariasis control programmes (which also use albendazole). Where the impacts of lymphatic filariasis control programmes on STH have been measured the impact on hookworm has been notable [30]. Another important route for adult treatment is through the treatment of women of reproductive age within other programmes – such as iron supplementation campaigns, but the level of treatment coverage is typically uncertain.

### Transmission control

#### Timeframe for elimination

Our projections indicate that targeting children alone does not notably impact the overall level of hookworm transmission (Fig. 3). Consequently, it is not possible to break transmission with drug administration alone without including the treatment of adults [8-10] (Fig. 4). These results are supported in part by a previous modelling study by Chan et al. [31], which investigated the effect of various mixing patterns between children and adults on the impact of hookworm treatment. Furthermore, in a recent field trial in an area with a low baseline hookworm infection, two semi-annual community-wide treatments with albendazole were enough to almost completely eliminate hookworm [30], which as such supports these model predictions. The level of treatment coverage in adults was the major determinant of the feasibility of eliminating hookworm transmission (Fig. 4). This finding contrasts with the model projections for *Ascaris* and *Trichuris*, where the coverage of children is often the major determinate [8-10], Turner* et al.*, ‘Analysis of the population-level impact of co-administering ivermectin with albendazole or mebendazole for the control and elimination of Trichuris trichiura’, submitted.

#### Cost savings

Due to the projected reductions in programme duration, it is possible for community-wide treatment to generate cost savings compared to the current child-targeted strategy. The extent of these costing savings was sensitive to the assumed increase in distribution costs when using community-wide treatment and the achieved coverage of adults (Table 5 and Additional file 1: Table S2). However, even when assuming it increases the programme’s distribution costs by 200 %, the analysis indicates potential cost savings in intermediate transmission settings when achieving a moderate to high coverage of adults (Table 5).

The higher the transmission setting and lower the coverage in adults, the lesser the possibility of cost savings from community-wide treatment (Fig. 5 and Table 5 and Additional file 1: Table S2). This occurs because the duration of community-wide treatment required to break transmission (Fig. 4), and therefore its total cost, increases with the intensity of the transmission setting (Fig. 5). However, it should be noted that these high transmission settings would have a higher prevalence of morbidity (Fig. 2). Therefore, though the cost savings of community-wide treatment are more uncertain in these settings, the benefits regarding morbidity and accelerating socioeconomic development will be more positive (Fig. 2).

### Costs of community-wide versus child-targeted treatment

Due to the absence of data, we calculated the distribution costs of community-wide treatment as a relative increase over the cost per year of child-targeted treatment. This reflects that the increase in cost for community-wide treatment will vary in different settings (see Methods).

It should be noted that it is often not possible to simply separate the costs of treating adults versus children – as they employ overlapping distribution systems [19]. For example, if many un-enrolled SAC or Pre-SAC are treated in the community rather than at the school, it would reduce the observed cost of community-based treatment (changing the apparent cost of treating adults). Consequently, the relative cost per treatment of the different strategies will also be influenced by the local demography (due to economies of scale) [19]. In addition, there will be fixed costs (i.e. those which do not depend on the number treated) shared between both distribution methods. Consequently, expanding treatment to target adults may generate economies of scale for the school-based programme, lowering the cost per treatment for children [19, 20, 32].

Better cost data will permit more detailed cost-effectiveness analysis, using absolute and not relative costs [19] and an understanding of how the costs may be influenced by coverage.

### Programmatic considerations of treating adults as well as children

Since the 2012 London Declaration on NTDs [33], the availability of donated drugs is no longer the major barrier to achieving the current WHO 2020 goals for STH control [34]. However, focus has now shifted to scaling up the implementation of STH programmes [34]. Expanding treatment programmes to include adults will require more programmatic resources and may not always be practical – particularly in countries which are struggling to achieve a high coverage of children. This highlights the fact that expanding treatment to communities will require further international aid support. However, experiences with lymphatic filariasis and onchocerciasis control programmes which use community-wide MDA clearly demonstrate that expansion is programmatically feasible and can be highly successful [35].

It is important to note that adding a community drug distributor (CDD) to treat adults would likely improve coverage of children (as the CDD may reach unenrolled children and Pre-SAC within the community more effectively). Where resources are limited to permit the expansion to community-wide treatment, a potential solution could be to further encourage the treatment of the parents of SAC within the school-based programmes. Ensuring the economic productivity of the children’s parents may also reduce school dropout rates.

GSK currently donates 400 million albendazole tablets each year for STH control [36]. Expanding programmes to target adults would increase the number of drugs doses needed considerably. This has important economic implications for donation programmes. However, it is important to acknowledge that the total amount of drugs required for donation could potentially decrease in the long term (due to the capacity to break transmission). Unfortunately, mebendazole is less effective against hookworm [14], so in this context programmes may have to rely on albendazole alone.

A further important programmatic consideration of treating continuously across entire communities is the potential risk of drug resistance developing. Restricting drug treatment to children creates a reservoir of untreated worms in adults that can effectively dilute any resistant gene pool in children. This issue needs careful monitoring with more research to define markers to track via molecular epidemiological studies.

### Limitations

Uncertainty still surrounds some aspects of hookworm biology and epidemiology. Consequently there is also uncertainty surrounding model structure and parameter estimation (and how these may differ for the different hookworm species). However, the results of this analysis depend largely on the observed age profiles of infection intensity and the observation that the adults harbour the majority of the worms – which was found to be robust across a range of different settings (Fig. 1a) and for both hookworm species (Fig. 1b); with the assumed host demography used within the model (from Uganda) [8], the proportion of worms harboured by adults was between 70 and 85 % for the different settings (Fig. 1). This range is also supported by [37]. It should be noted that the potential cost-savings were even more pronounced when using an alternative dataset for the baseline age profile of infection intensity (Fig. 1 and Additional file 1: Table S4). They were also robust when changing the parameter for the strength of the density dependence in egg production by female worms (Additional file 1: Table S3).

Within the model, a worm burden above previously established intensity thresholds (Table 1) was used as a proxy for morbidity [15]. It should be acknowledged that these thresholds (Table 1) are uncertain and are likely to be influenced by a number of host specific factors [38, 39]. Furthermore, the thresholds were based on a study where *N. americanus* was the predominant hookworm species and may be too high for *A. duodenale* (which is associated with higher rates of blood loss per worm) [16]. More research is needed for statistical models that relate the disease burden of hookworm to experience of infection and the impact of treatment, to allow models to accurately estimate a cost per DALY averted.

This analysis assumed that once hookworm transmission is broken the costs of control stop immediately. However, in reality, certifying elimination would require a period of post-intervention surveillance with associated costs. Furthermore, STH control programmes also target *Ascaris* and *Trichuris* and therefore may need to be continued even if hookworm is eliminated (though community-wide treatment would also accelerate the timeframe for breaking transmission for these infections as well [8, 9, 10]).

The model does not currently address the possibility of systematic non-compliance. If a high proportion of adults were systematically non-compliant, the timeframe for elimination would be increased, reducing the potential cost savings of community-wide treatment (though the general principles still apply).

## Conclusions

Annual community-wide treatment is predicted to be markedly more cost-effective in controlling both hookworm’s disease burden and the level of on-going transmission. Furthermore, because it is possible to break transmission when using community-wide treatment (which reduces the required programme duration), expanding programmes can generate long term cost savings– even if it notably increases the annual distribution costs of the programme when achieving a moderate to high coverage of adults.

Although expanding programmes to target adults will require greater programmatic resources and drug donations, the calculations highlight the notable benefits it can generate and the importance of further considering this strategy in any ongoing revisions of the treatment and control guidelines for STH. These results highlight that the current WHO guidelines, which recommend treating all STH the same way (regardless of which age groups have the highest burden), may lead to an inefficient use of available drug donations and associated resources.

## Additional file

Additional file 1:
**Supporting information.** (DOCX 762 kb)
