# Supplementary material for: An economic evaluation of expanding hookworm control strategies to target the whole community
Source: Parasit Vectors. 2015 Nov 5;8:570. doi: 10.1186/s13071-015-1187-5 (PMC4635541; doi:10.1186/s13071-015-1187-5)
Supplement: Additional file 1: — Supporting information. (DOCX 762 kb) [file 13071_2015_1187_MOESM1_ESM.docx]

**Supporting information**

**The Model**

The fundamental model used to describe the mean worm burden of individuals of a given age and the quantity of infectious eggs in the environment is taken from Anderson and May [[1](#_ENREF_1)]. The current version of the model is described in detail in [[2](#_ENREF_2), [3](#_ENREF_3)].

## For the purposes of this analysis the models output, which is in terms of the mean number of female worms [[2](#_ENREF_2), [3](#_ENREF_3)], was converted to the mean number of worms (both males and females) assuming a 1:1 sex ratio [[4](#_ENREF_4), [5](#_ENREF_5)] i.e. the modelled female worm burden output was doubled.

In this paper we used the model parameters pertaining to Hookworm*,* described in Table S1.

| **Supporting Table S1: Model parameters for Hookworm** | |  |
| --- | --- | --- |
| **Parameter** | **Value** | **Source** |
| Adult worm life expectancy (years) | 2 | [[1](#_ENREF_1)] |
| Density dependence acting fecundity (egg production by female worms),$z$ | 0.92 | Fitted to the data in [[6](#_ENREF_6)] |
| Aggregation parameter, *k* | 0.35 | [[6](#_ENREF_6)] |
| Life expectancy of the infective stage | 10 days | [[1](#_ENREF_1)] |
| Relative values for the degree of exposure, *β,* and contribution, *ρ,* of the various age groups to the infectious reservoir | 0-2 year olds = 0.03,  2-5 year olds = 0.09,  5-15 year olds = 1,  15+ year olds = 2.5 | Fitted to the data in [[6](#_ENREF_6)] |
| Proportion of humans at age *a*, $P(a)$ | Based on Uganda’s demographical profile | [[3](#_ENREF_3), [7](#_ENREF_7)] |

**Supporting Figure S1: Model fit to cross-sectional data from the Zimbabwe [**[**6**](#_ENREF_6)**].** *Individual data points pertain to the mean total worm burden reported by* [[6](#_ENREF_6)]*. The solid line is equilibrium mean worm burden with age given by the model.*


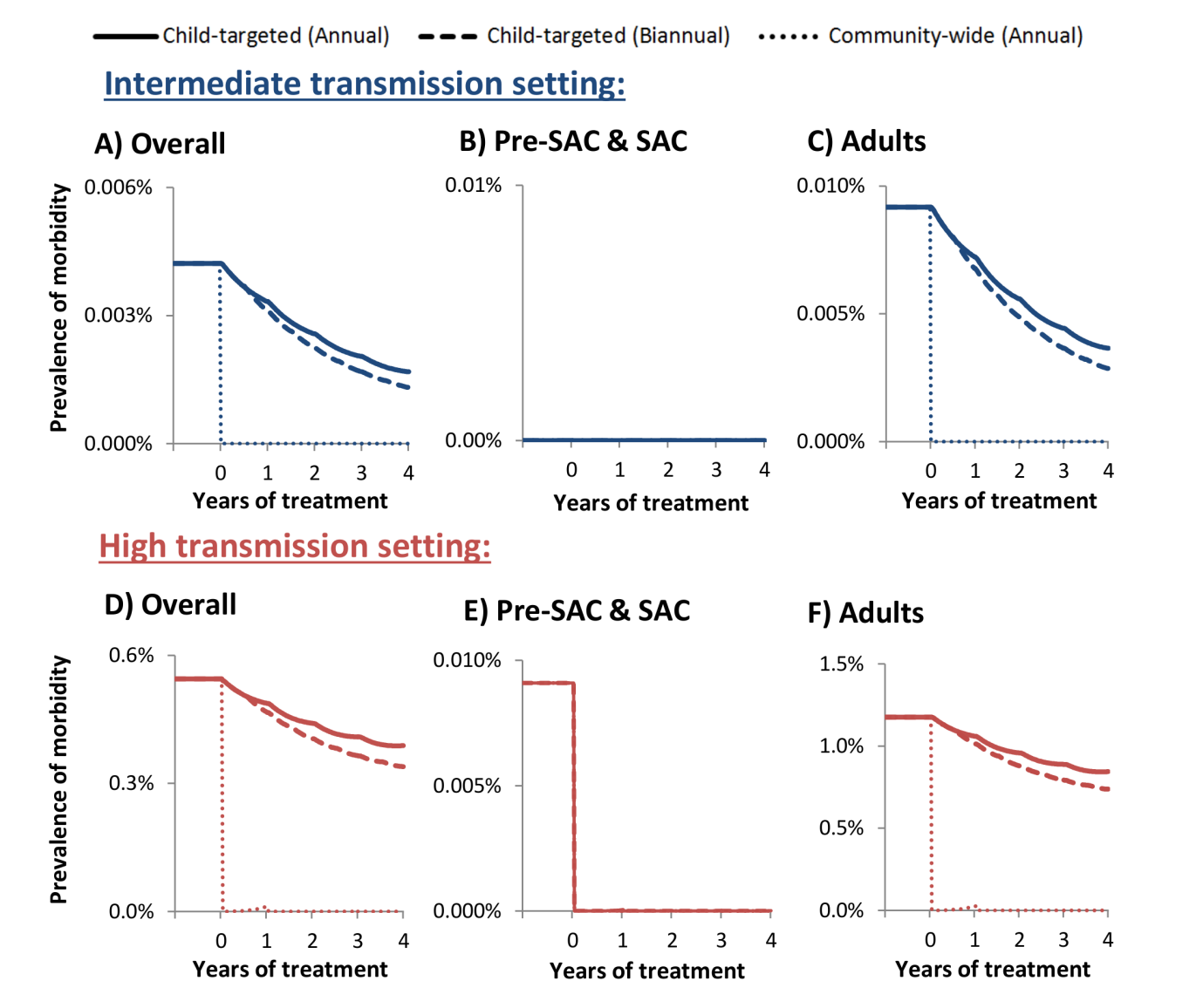
**Supporting Figure S2: Impact of different treatment strategies on the prevalence of morbidity in A) the overall community, B) Pre-SAC & SAC and C) adults.** *The results employ the higher intensity thresholds for morbidity (presented in Table 1).Two different transmission settings were explored; intermediate (R_0_=2.5), and high (R_0_=5) – as measured by the basic reproductive number (R_0_) [*[*8*](#_ENREF_8)*]. Panels A & D illustrate the overall mean number of worms across all ages, panels B & E, the mean number of worms in children (Pre-SAC and SAC, 2-14 year olds), panels C & F record the mean number of worms in adults (≥15 year olds). The different styled lines represent different treatment strategies: solid – annual targeted treatment (Pre-SAC and SAC), dashed – biannual targeted treatment (Pre-SAC and SAC), and dotted – annual community-wide treatment (Pre-SAC, SAC and adults). Individuals under two years of age were not eligible for treatment. The results assume 80% coverage per round of targeted age group(s), and 94.8% treatment efficacy. The corresponding results using the lower intensity threshold are presented in Figure 2 of the main text.*

*
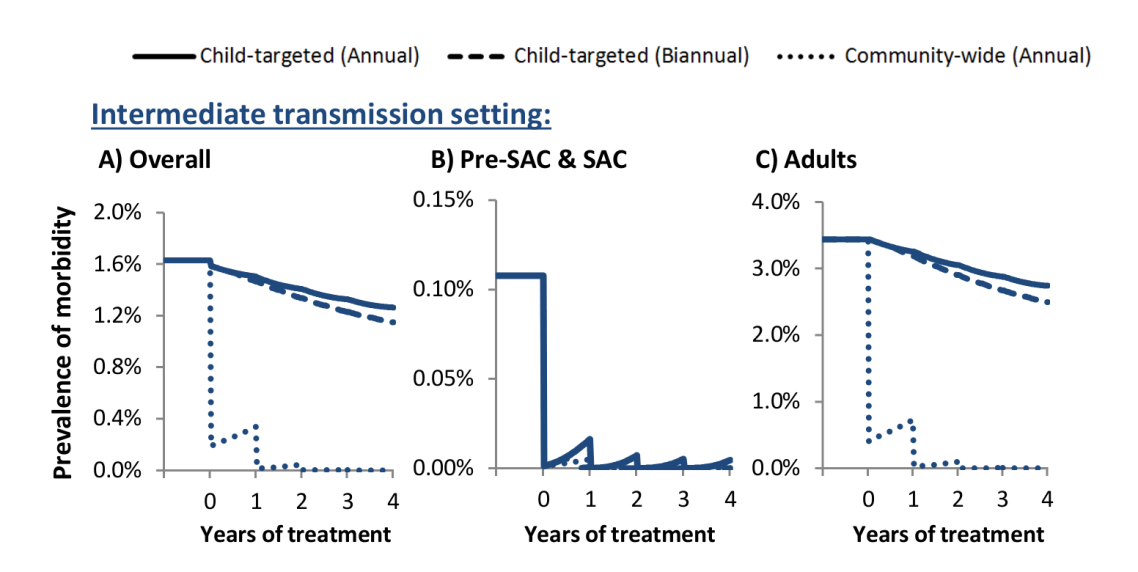
***Supporting Figure S3: Impact of different treatment strategies on the prevalence of morbidity in A) the overall community, B) Pre-SAC & SAC and C) adults.** *The results employ the lower intensity thresholds for morbidity (presented in Table 1). Panel A illustrate the overall mean number of worms across all ages, panel B the mean number of worms in children (Pre-SAC and SAC, 2-14 year olds), panel C the mean number of worms in adults (≥15 year olds). The different styled lines represent different treatment strategies: solid – annual targeted treatment (Pre-SAC and SAC), dashed – biannual targeted treatment (Pre-SAC and SAC), and dotted – annual community-wide treatment (Pre-SAC, SAC and adults). Individuals under two years of age were not eligible for treatment. The results assume, intermediate (R_0_=2.5) transmission setting, 80% coverage per round of targeted age group(s), and 64.2% treatment efficacy.*


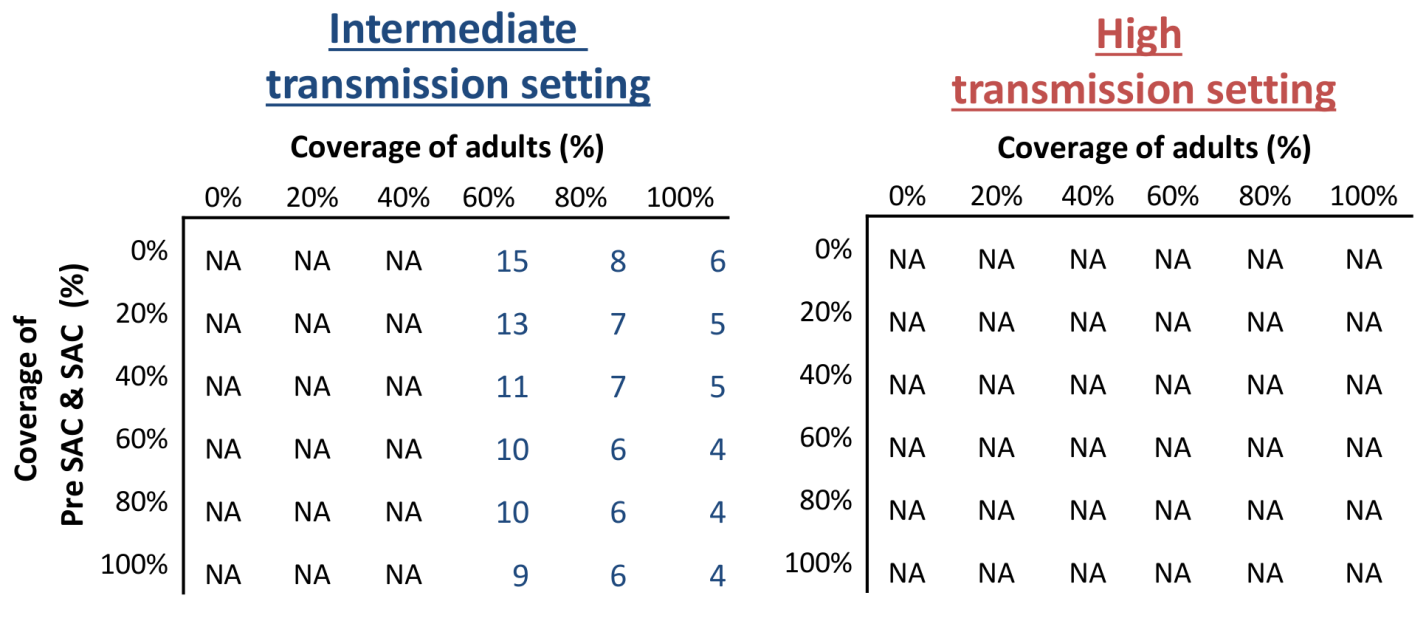
**Supporting Figure S4: Number of years of annual treatment to achieve elimination of hookworm as a function of coverage of children versus adults**. *Two different transmission settings were explored; intermediate (R_0_=2.5), and high (R_0_=5)– as measured by the basic reproductive number (R_0_) [*[*8*](#_ENREF_8)*]. Results assume a 64.2% treatment efficacy. Pre-SAC and SAC, 2-14 year olds and adults, ≥15 year olds. Individuals under two years of age were not eligible for treatment. NA; Not achievable within 15 years of annual treatment.*

| **Supporting Table S2: The relative total cost of annual community-wide versus child-targeted treatment** | | | | |
| --- | --- | --- | --- | --- |
|  | **Relative increase in the distribution costs (total per year) of community-wide versus child-targeted treatment** | | | |
|  | **+50%** | **+100%** | **+150%** | **+200%** |
| **Time horizon** |  |  |  |  |
| **Coverage of adults: 75%** | | | | |
| **Intermediate transmission** | |  |  |  |
| 20 years | -71% | -62% | -52% | -43% |
| 35 years | -80% | -74% | -67% | -60% |
| 50 years | -83% | -78% | -72% | -67% |
| **High transmission setting** | |  |  |  |
| 20 years | -21% | **+5%** | **+31%** | **+57%** |
| 35 years | -46% | -28% | -9% | **+9%** |
| 50 years | -55% | -39% | -24% | -9% |
| **Coverage of adults: 55%** | | | | |
| **Intermediate transmission** | |  |  |  |
| 20 years | -45% | -27% | -6% | **+9%** |
| 35 years | -62% | -50% | -35% | -24% |
| 50 years | -68% | -58% | -45% | -37% |
| **High transmission setting** | |  |  |  |
| 20 years | NA | NA | NA | NA |
| 35 years | NA | NA | NA | NA |
| 50 years | NA | NA | NA | NA |
| **Coverage of adults: 35%** | | | | |
| **Intermediate transmission** | |  |  |  |
| 20 years | **+1%** | **-+4%** | **+68%** | **+101%** |
| 35 years | -30% | -7% | **+16%** | **+39%** |
| 50 years | -41% | -23% | -3% | **-16%** |
| **High transmission setting** | |  |  |  |
| 20 years | NA | NA | NA | NA |
| 35 years | NA | NA | NA | NA |
| 50 years | NA | NA | NA | NA |
| *The results assume an annual treatment strategy, 75% treatment coverage of children, and 94.8% treatment efficacy. Two different transmission settings were explored; intermediate (R_0_=2.5), and high (R_0_=5). Costs were discounted at 3% per year. Results in bold are where the total costs of community-wide treatment are higher than child-targeted treatment (i.e. it was not cost saving and has a higher relative cost). * Elimination not achievable within 15 year (and therefore there are no reductions in programme duration).* | | | | |

| **Supporting Table S3: Sensitivity of the relative total cost of annual community-wide versus child-targeted treatment to the** **strength of the** **density dependence in egg production by female worms** | | | | |
| --- | --- | --- | --- | --- |
| **Intermediate transmission setting** | **Relative increase in the distribution costs (total per year) of community-wide versus child-targeted treatment** | | | |
|  | **+50%** | **+100%** | **+150%** | **+200%** |
| **Time horizon** |  |  |  |  |
| **Coverage of adults: 75%** | | | | |
| 20 years | -62%, -71% | -50%,- 62% | -34%, -52% | -25%, -43% |
| 35 years | -74%, -80% | -65%, -74% | -54%, -67% | -48%, -60% |
| 50 years | -78%, -83% | -71%, -78% | -62%,- 72% | -57%, -67% |
| *The results assume an annual treatment strategy, 75% treatment coverage of children, and 94.8% treatment efficacy. Range compares the credible intervals for the strength of the density dependence in egg production by female worms (z =0.87-0.95). R_0_ was varied so that the initial worm burden was the same across the different scenarios. Costs were discounted at 3% per year.* | | | | |

| **Supporting Table S4: Sensitivity of relative total cost of annual community-wide versus child-targeted treatment to an alternative baseline age-intensity profile** | | | | |
| --- | --- | --- | --- | --- |
| **Intermediate transmission setting** | **Relative increase in the distribution costs (total per year) of community-wide versus child-targeted treatment** | | | |
|  | **+50%** | **+100%** | **+150%** | **+200%** |
| **Time horizon** |  |  |  |  |
| **Coverage of adults: 75%** | | | | |
| 20 years | -71%, -71% | -62%, -62% | -52%, -52% | -43%, -43% |
| 35 years | -80%, -80% | -74%, -74% | -67%, -67% | -60%, -60% |
| 50 years | -83%, -83% | -78%, -78% | -72%, -72% | -67%, -67% |
| **Coverage of adults: 55%** | | | | |
| 20 years | -54%, -45% | -38%, -27% | -20%, -6% | -7%, **+9%** |
| 35 years | -68%, -62% | -57%, -50% | -44%, -35% | -36%, -24% |
| 50 years | -73%, -68% | -64%, -58% | -54%, -45% | -47%, -37% |
| **Coverage of adults: 35%** | | | | |
| 20 years | -29%, **+1%** | -5%, **+34%** | **+18%, +68%** | **+42%, +101%** |
| 35 years | -51%, -30% | -35%, -7% | -18%, **+16%** | -2%, **+39%** |
| 50 years | -59%, -42% | -45%, -23% | -32%, -3% | -18%, **+16%** |
| *The results assume an annual treatment strategy, 75% treatment coverage of children, an R_0_ of 2.5 and 94.8% treatment efficacy. Range compares the results from the fitted baseline age-intensity profile (Figure S1) to an alternative fit to Anderson et al. [*[*9*](#_ENREF_9)*] (N. americanus). This profile was more conservative and had one of the lowest percentage worm burden in adults (Figure 1). The β for the new fit are; 0-4 year olds = 0.01, 5-8 year olds =0.14, 9-19 year olds =1, 20-70 year olds = 0.62. Costs were discounted at 3% per year. Results in bold are where the total costs of community-wide treatment are higher than child-targeted treatment (i.e. it was not cost saving and has a higher relative cost).* | | | | |

| **Supporting Table S5: Sensitivity of the relative total cost of community-wide versus child-targeted treatment to the assumed discount rate.** | | | | |
| --- | --- | --- | --- | --- |
|  | **Relative increase in the distribution costs (total per year) of community-wide versus child-targeted treatment** | | | |
|  | **+50%** | **+100%** | **+150%** | **+200%** |
| **Discount rate** |  |  |  |  |
| **Coverage of adults: 75%** | | | | |
| **Intermediate transmission** | |  |  |  |
| 0% | -87% | -83% | -77% | -74% |
| 3% | -80% | -74% | -65% | -60% |
| 6% | -72% | -63% | -51% | -45% |
| **High transmission setting** | |  |  |  |
| 0% | -32% | -10% | **+13%** | **+35%** |
| 3% | -16% | **+12%** | **+40%** | **+68%** |
| 6% | **+1%** | **+35%** | **+68%** | **+102%** |
| *The results assume an annual treatment strategy, 75% coverage per round of targeted age group(s), and 94.8% treatment efficacy. Results assume a 20 year time horizon. Two different transmission settings were explored; intermediate (R_0_=2.5), and high (R_0_=5). Results in bold are where the total cost of community-wide treatment is higher than child-targeted treatment.* | | | | |

**References**

1. Anderson RM, May RM: **Helminth infections of humans: mathematical models, population dynamics, and control**. *Adv Parasitol* 1985, **24**:1-101.

2. Truscott JE, Hollingsworth TD, Brooker SJ, Anderson RM: **Can chemotherapy alone eliminate the transmission of soil transmitted helminths?** *Parasit Vectors* 2014, **7**(1):266.

3. Anderson RM, Truscott JE, Hollingsworth TD: **The coverage and frequency of mass drug administration required to eliminate persistent transmission of soil-transmitted helminths**. *Philos Trans R Soc Lond B Biol Sci* 2014, **369**(1645):20130435.

4. Anderson RM: **The population dynamics and control of hookworm and roundworm infections**. In: *The Population Dynamics of Infectious Diseases: Theory and Applications.* Edited by Anderson RM: Springer US; 1982: 67-108.

5. Guyatt HL, Bundy DA: **Estimation of intestinal nematode prevalence: influence of parasite mating patterns**. *Parasitology* 1993, **107 (Pt 1)**:99-105.

6. Bradley M, Chandiwana SK, Bundy DA, Medley GF: **The epidemiology and population biology of *Necator americanus* infection in a rural community in Zimbabwe**. *Trans R Soc Trop Med Hyg* 1992, **86**(1):73-76.

7. Pullan RL, Kabatereine NB, Quinnell RJ, Brooker S: **Spatial and genetic epidemiology of hookworm in a rural community in Uganda**. *PLoS Negl Trop Dis* 2010, **4**(6):e713.

8. Anderson RM, May RM: **Infectious diseases of humans: dynamics and control** In*.* Oxford: Oxford Science Publications; 1991.

9. Anderson RM, Schad GA: **Hookworm burdens and faecal egg counts: an analysis of the biological basis of variation**. *Trans R Soc Trop Med Hyg* 1985, **79**(6):812-825.
